# Influences of temporal order in temporal reproduction

**DOI:** 10.3758/s13423-023-02310-5

**Published:** 2023-06-08

**Authors:** Cemre Baykan, Xiuna Zhu, Fredrik Allenmark, Zhuanghua Shi

**Affiliations:** General and Experimental Psychology, Department of Psychology, Ludwig Maximilian University of Munich, 80802 Munich, Germany

**Keywords:** Time perception, Temporal reproduction, Temporal order, Temporal sequence perception, Auditory pattern reproduction

## Abstract

Despite the crucial role of complex temporal sequences, such as speech and music, in our everyday lives, our ability to acquire and reproduce these patterns is prone to various contextual biases. In this study, we examined how the temporal order of auditory sequences affects temporal reproduction. Participants were asked to reproduce accelerating, decelerating or random sequences, each consisting of four intervals, by tapping their fingers. Our results showed that the reproduction and the reproduction variability were influenced by the sequential structure and interval orders. The mean reproduced interval was assimilated by the first interval of the sequence, with the lowest mean for decelerating and the highest for accelerating sequences. Additionally, the central tendency bias was affected by the volatility and the last interval of the sequence, resulting in a stronger central tendency in the random and decelerating sequences than the accelerating sequence. Using Bayesian integration between the ensemble mean of the sequence and individual durations and considering the perceptual uncertainty associated with the sequential structure and position, we were able to accurately predict the behavioral results. The findings highlight the critical role of the temporal order of a sequence in temporal pattern reproduction, with the first interval exerting greater influence on mean reproduction and the volatility and the last interval contributing to the perceptual uncertainty of individual intervals and the central tendency bias.

## Introduction

Precise timing is critical in diverse everyday activities, including social interactions and adaptive motor behaviors. Accurate processing of temporal patterns enables us to discern subtle differences in conversation or musical rhythms (London et al., 2016; Wang et al., 2021). Studies indicate that humans are proficient at discerning complex rhythmic patterns in both visual and auditory domains (Grahn, 2012; Su & Salazar-López, 2016). However, contextual modulation and integration affect perception of temporal patterns, especially in complex real-world scenarios involving multiple rhythmic cues.

In a simple rhythmic form, changes in auditory click rate can assimilate the apparent flicker rate of a flashing light, known as the auditory driving effect (Recanzone, 2003; Shipley, 1964; Welch et al., 1986). This effect is thought to occur due to cross-modal interactions in the brain. The classic time-shrinking illusion, where a train of different intervals assimilate to each other (Nagaike et al., 2016; Nakajima et al., 1992), further demonstrates how intervals can be perceived as shorter or longer based on their preceding intervals. Studies on rhythm adaptation show similar effects, such as adapting to faster rhythms leading to shorter perception of the following sequence (Becker & Rasmussen, 2007) and decelerating rhythms leading to perception of the following isochronous rhythm as accelerating (Li et al., 2022).

Despite extensive research on temporal rhythms, the precise reproduction of temporal structure in terms of time intervals has yet been largely overlooked. The perception and reproduction of temporal sequences depend not only on the individual time intervals but also on the perception of the rhythm itself, which is affected by temporal order and the relative length of its subcomponents (Matthews, 2013). Reproducing individual elements of a group can be challenging, as demonstrated in a study of size estimation (Ariely, 2001), where identifying individual items was difficult but the average size could be estimated accurately. The ability to extract the summary statistics of a set is called ensemble perception (for a review, see Whitney & Yamanashi Leib, 2018).

Ensemble perception can make it difficult to recall or reproduce individual items in a set, as ensemble statistics may heavily influence perception of individual items (Ariely, 2001; Whitney & Yamanashi Leib, 2018). Zhu et al. (2021), for example, found that judgments of individual intervals in a bisection task were assimilated to the mean of the probed intervals across trials, showing a central tendency effect (Jazayeri & Shadlen, 2010; Laming, 1999). Previous studies on the time-shrinking illusion (Burr et al., 2013; Nagaike et al., 2016; Nakajima et al., 1992) suggest that such assimilation may occur within a sequence of intervals within a trial. However, these studies did not examine the stimulus order effect within temporal sequences.

The order effect within a trial has been investigated in a simple two-interval comparison. For example, the threshold and the discrimination sensitivity depend on the order of the standard and the comparison, known as time-order error (TOE; Allan, 1977; Dyjas et al., 2012; Hellström, 1985; Ulrich & Vorberg, 2009). The discrimination sensitivity is higher when the standard is presented first. The order effect is not limited to stimuli presented within individual trials: Sequential order of stimuli across trials can also systematically alter judgments (Glasauer & Shi, 2019, 2021, 2022; Shi et al., 2022). For example, Glasauer and Shi (2021) tested reproduction of the same set of intervals in two different sequential structures: random walk and completely random order. The random-walk sequence exhibits mild fluctuation of successive intervals, while the random sequence has unpredicted fluctuations. The former is analogous to temperature fluctuations occurring over consecutive days, while the latter is like the random rearranged temperature data from a year. Reproduction in a random sequence, relative to a random walk sequence, yielded a pronounced central tendency effect, suggesting that the volatility^1^ of the sequence can influence how an interval is reproduced. However, these studies, including the study of Glasauer and Shi (2021), have only used single interval reproduction. Timing of isolated intervals (i.e., interval timing) and complex pattern timing involve different neural circuits of the brain (Teki et al., 2011), leaving unanswered the question of how the order of a temporal sequence influences its reproduction.

On these backgrounds, we aimed to investigate the effect of sequential structure, particularly the order (and the volatility) of sequences in temporal reproduction. We hypothesized that the perceived volatility of a sequence may affect the ensemble representation of the sequence, and subsequently influence its reproduction. In addition to the order of intervals, the first and the last intervals in a sequence may also introduce additional primacy and recency effects, respectively (Deese & Kaufman, 1957). Primacy and recency effects are two phenomena related to memory and information processing that describe the tendency of better remembering the first and last item in a list (Silverman, 2010). The first interval is often perceived as longer than subsequent intervals because of the onset saliency of the first interval (Kanai et al., 2006; Rose & Summers, 1995). According to Bayesian inference of timing (e.g., Shi, Church, et al., 2013a), perception tends to assimilate towards a more reliable source. As people are better at recalling the endpoints (the first and the last intervals) of a rhythmic pattern, they are more likely to depend on these endpoints for perceiving and reproducing a rhythm. Consequently, rhythms with more variable endpoints are expected to have a higher central tendency.

To disentangle these effects, we conducted a rhythm production task where participants reproduced a series of auditory stimuli presented in succession. The temporal patterns we used had the same mean and variance, but differed in their structure: decelerating (DS), accelerating (AS), or random (RS). We expected the sequential structure, the first, and the last interval to have great impact due to volatility, the primacy and recency effects.

## Method

### Participants

Fifteen participants (seven females, mean age = 25.3 years) with normal hearing took part in the experiment at Ludwig Maximilian University (LMU) of Munich. The sample size was determined based on the sample sizes of previous similar temporal pattern reproduction studies (Hardy et al., 2018; Laje et al., 2011), in which 11 to 12 participants were recruited. We further increased the sample size to 15 to ensure the statistical power of the study. Written informed consent was received from all participants before the experiment. They received 9 Euro per hour or course credit for their participation. The study protocol was approved by the Ethics Board of the Department of Psychology at LMU Munich. All participants were naive to the purpose of the research.

### Apparatus and stimuli

The experiment was conducted in a sound-reduced and moderately lit testing room. Instructions and feedback were presented on a CRT monitor. Auditory stimuli were generated with customized MATLAB codes and presented via Sony MDR stereo headphones using the Psychtoolbox-3 (Kleiner et al., 2007). Responses were acquired via a computer mouse for the reproduction task. In the experiment, three temporal patterns were compared: the decelerating sequence (DS), accelerating sequence (AS), and random sequence (RS). Each sequence had four intervals demarcated by five ‘beep’ sounds. A decelerating sequence structure denotes a pattern of intervals in descending rhythmic order (from short to long), whereas an accelerating sequence structure has a reserve pattern. For a random temporal pattern, time intervals were presented in a random order excluding decelerating or accelerating structures. Thus, the random temporal pattern had the highest volatility.

**Fig. 1:**
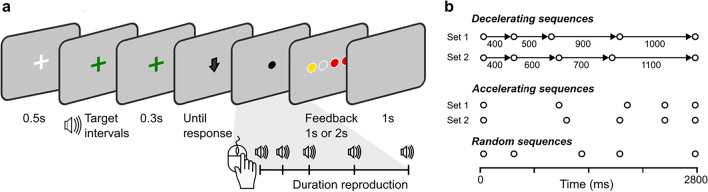
Illustration of the experimental design and target intervals. **a** Each trial started with a fixation cross presented for 500 ms in the middle of the screen. Participants received five beep sounds marking the four sequential intervals, followed by a 300-ms-long presentation of the fixation cross. Then, they were asked to reproduce the temporal pattern by clicking the mouse. After the reproduction, a feedback was shown to indicate the accuracy of the reproduction (see the main text in Procedure for more details). After a blank period of 1,000 ms, the next trial began. **b** Illustration of the temporal patterns used in the experiment. Each circle represents a beep sound. Two sets of intervals were tested in the decelerating (DS) and accelerating (AS) sequences. The AS condition consisted of the same interval sets used in the DS condition but the intervals were in the inverted order, whereas the RS condition consisted of the same intervals randomized (in the illustration, only one possible sequence is shown). (Color figure online)

In each sequence type, we used two interval sets that were randomly shuffled across trials to avoid participants recognizing the four fixed intervals. In the DS condition, two sets of intervals with the same mean (700 ms) and the standard deviation (*SD* = 294.39 ms) were tested: [400, 500, 900, 1,000] ms and [400, 600, 700, 1,100] ms, whereas in the AS condition, these two interval sets were in the inverted order (Fig. 1b). In the RS condition, the orders of the sample intervals were randomized, and all four-intervals ascending or descending orders were excluded to distinguish them from the other two conditions. Thus, in all sequences, the first and the second moments of the ensemble statistics were the same (*M* = 700 ms, *SD* = 294.39 ms).

To present the intervals accurately and precisely, we created sound waves of five brief beeps (20 ms, 1000 Hz, 60 dB) for each stimulus-onset asynchrony (SOA) that corresponded to one of the four intervals. The sound wave lasted for a duration of 2.8 seconds.

### Procedure

The task was to reproduce a sequence of auditory time intervals, presented within a single trial, using mouse clicks. As illustrated in Fig. 1a, each trial started with a white fixation cross for 500 ms. Next, as the fixation cross turned to green color, participants heard the sequence of five consecutive beeps that demarcated four temporal intervals. The total duration of the intervals was 2.8 seconds, which was immediately followed by a gap of 300 ms. A down-arrow image then appeared on the screen to indicate the reproduction of the temporal pattern could be started. The reproduction was performed by clicking the mouse button five times, each mouse click producing a beep, and attempting to as closely as possible imitate the previously heard sequence of beeps. Each button press initiated a beep for 20 ms (1000 Hz, 60 dB) regardless of the pressing duration. After the reproduction, a feedback display was shown for accuracy. The feedback consisted of four adjacent circles showing how close the participants’ reproduction to the veridical interval of each sample interval was. The four disks represented the four sequential intervals from the left to the right, and their colors indicated correspondent reproduced accuracy. The red disk indicated the relative error was 50% longer or shorter than the sample duration, and the yellow disk for the relative error in between [−50%, −15%] or [15%, 50%], the white disk filled with gray color (as it was the same as the background color of gray) for the relative error in between [−15%, 15%]. To encourage participants to reproduce the intervals accurately, the duration of the feedback display was contingent on the accuracy. If more than three reproduced intervals were in ‘red’ or ‘yellow’, the feedback display lasted for two seconds, otherwise, it was shown only for 1 second. After a 1-second blank screen, the next trial started. Participants received one practice block of 44 trials prior to the main experiment to familiarize them with the task, which was discarded in the formal analysis. The main experiment consisted of six blocks, with 44 trials in each block, and 264 trials in total. The orders of the trials were randomized for each participant.

### Statistical analyses

All statistical tests were carried out in either Python or JASP. Repeated-measures analyses of variance (ANOVAs) were used for factorial analyses. We further conducted Bayesian ANOVAs with default settings (i.e., r-scale fixed effects = 0.5, r-scale random effects = 1, r-scale covariates = 0.354) to provide a more rigid criteria required for hypothesis testing (Kass & Raftery, 1995; Rouder et al., 2009). All Bayes factors reported for the main effects and interactions were calculated using “inclusion” Bayes factors across matched models. Given that we report standard significance, Bayes factors above 10 for significant results are often superfluous. Therefore, we only report them when they are less than 10. Post hoc *t* tests were Bonferroni corrected. Additionally, outliers were excluded from all the statistical analyses below. These outliers were defined as trials on which the reproduced interval mean of the stimulus sequence differed by more than two times the standard deviation of the sequence mean, which accounted for approximately 3% of all trials.

### Modeling

As demonstrated in the behavioral results below, the mean reproduced interval was influenced by the first probe interval. Therefore, in our model, we assumed the mean prior (*μ*_*e*_) of the sequence is a weighted average of the first probe interval (*D*_1_) and the mean of the tested intervals (700 ms):

1 $${\mu}_e=\alpha \cdot {D}_1+700\cdot \left(1-\alpha \right),$$

with the weight *α* being determined by the variability of the stimulus distribution and measurement noise. Since duration estimates often follow Weber’s law (Shi, Church, et al., 2013a), the sensory variability and the prior variability are determined by Weber fractions (*wf*_*s*_ and *wf*_*p*_ respectively). In addition, we assumed the perceived volatility of the sequence may scale the sensory uncertainty. To be more precise, the variability (*σ*_*i*_) of a given interval *D*_*i*_ has a volatility factor *k*_*j*_, which depends on the sequence type *j*,

2 $${\sigma}_i^2={k}_j{\left(w{f}_s\cdot {D}_i\right)}^2.$$

The reproduction pattern (*R*_*i*_) is then modeled as Bayesian integration between the ensemble mean *μ*_*e*_ and individual duration *D*_*i*_ (Jazayeri & Shadlen, 2010; Ren et al., 2021; Shi, Church, et al., 2013a):

3 $${R}_i=\left(1-w\right){\mu}_e+w\ {D}_i,$$

where weight $w\propto 1/{\sigma}_i^2$, the reproduction variance ${\sigma_r}^2=\frac{\left({\sigma}_i^2.{\sigma}_e^2\right)}{\left({\sigma}_i^2+{\sigma}_e^2\right)}$, and *σ*_*e*_ = *wf*_*p*_ · *μ*_*e*_ . This integration yields a central tendency bias. That is, intervals shorter than the ensemble mean *μ*_*e*_ are overestimated, and intervals longer than the mean are underestimated. To measure this bias, a linear regression was conducted between sample intervals and reproduced intervals.

Based on these assumptions, we fitted the model to the observed data and estimated duration reproduction patterns for each participant. The model was implemented using PyMC3 (Salvatier et al., 2016). To demonstrate the robustness and predictiveness of the model, we first fit the model with the structured sequences (i.e., AS and DS) and then use those fitted parameters (excluding the sequence scaling factor *k*) for the RS condition. We also assessed the model’s goodness using coefficients of determination (*R*^2^) between the predicted and observed data.

## Results

### Effects of the temporal pattern on the central tendency bias

**Fig. 2:**
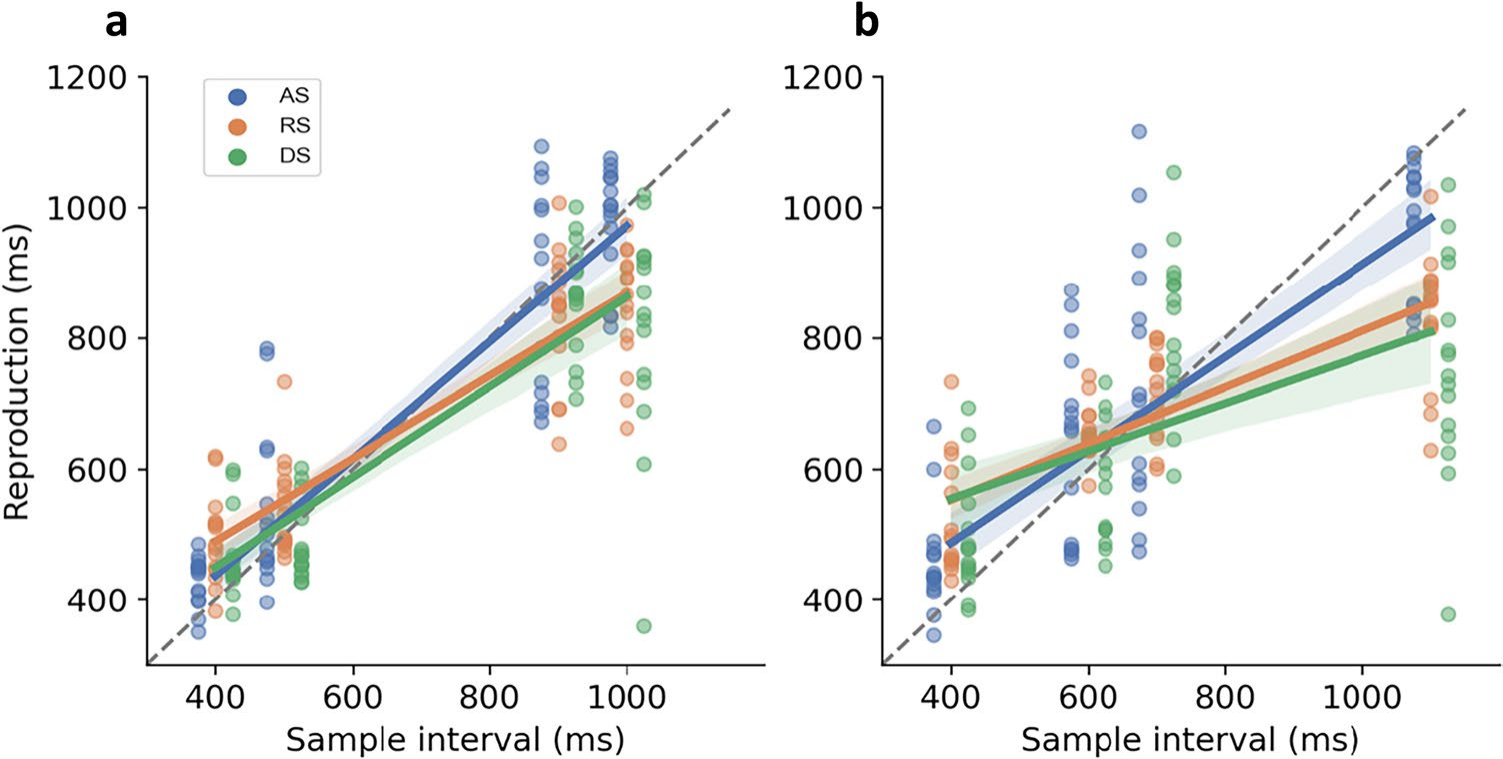
The slope of the reproduction lines varied as a function of stimuli sequences. The AS condition (blue) had a significantly steeper reproduction line than the RS (orange) and the DS (green) conditions. The DS condition showed the most deviations from the equal reproduction line for which the subjective reproduction would be identical to the sample intervals (diagonal dashed lines) towards the means of the temporal patterns (700 ms). Each dot on a column of sample intervals corresponds to the data point of one single participant. Dots shifted gradually by conditions for illustration purposes. **a** Reproductions of Interval Set 1: type of pattern created using 400, 500, 900, and 1,000 ms intervals. **b** Reproductions of Interval Set 2: type of pattern created using 400, 600, 700, and 1,100 ms intervals. (Color figure online)

As depicted in Fig. 2, all three sequence types exhibited the central tendency bias, with reproduction slopes less than one (*M* = 0.80, 0.526, 0.53 for the AS, DS, and RS, respectively). A two-way repeated-measures ANOVA revealed significant main effects of sequence type, *F*(2, 28) = 16.92, *p* < .001, ${\eta}_p^2=.55$, and interval set, *F*(1, 14) = 57.93, *p* < .001, ${\eta}_p^2=.81$. Post hoc comparisons revealed that AS had a steeper slope (i.e., less central tendency) compared with RS, *t*(14) = 6.50, *p* < .001, and DS, *t*(14) = 3.85, *p <* .01. However, there were no significant differences between the RS and DS, *t*(14) = 0.09, *p* = .93, *BF* = 0.26. The significant difference between the two interval sets suggests that the separation of individual intervals may also affect reproduction (as seen by the horizontal gaps among points in Fig. 2). An interaction between interval set and sequence type was also observed, *F*(2, 28) = 4.00, *p* = .03, ${\eta}_p^2$= .22, *BF* = 0.51. However, the small BF value suggests that this interaction should not be overinterpreted.

### Influences of the first and the last intervals

**Fig. 3:**
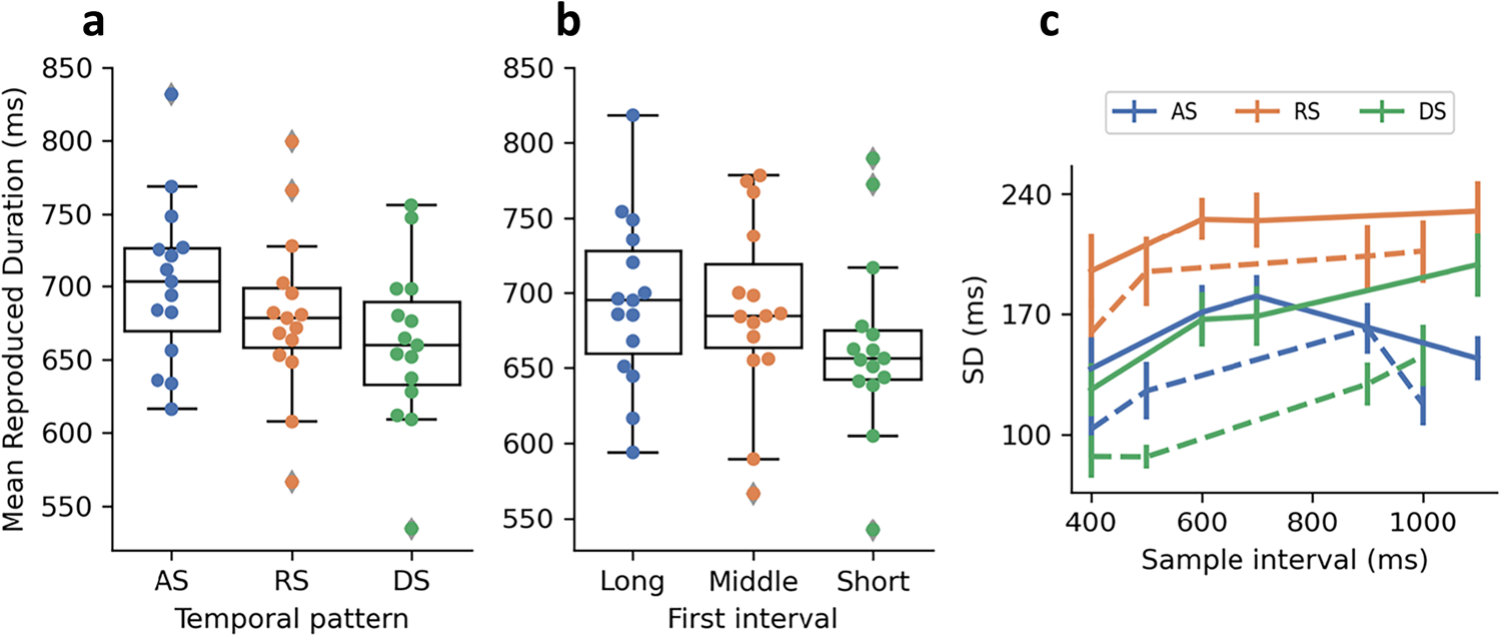
**a** Boxplots of the mean reproduction of sequence intervals (AS: accelerating sequences; RS: random sequences; DS: decelerating sequences). The dots depict the mean reproductions of one participant for one sequence type, averaged over Interval Set 1 and Set 2. **b** Boxplots of the mean reproduction of sequence intervals in the RS, divided into three categories based on the time interval in the initial position (Long: first interval longer than 700 ms; Middle: first interval of 700 ms; Short: first interval shorter than 700 ms). The dots depict the mean reproductions of one participant for one sequence type, averaged across both interval sets. **c** Standard deviation of reproductions in the AS (blue), RS (orange) and DS (green) conditions. Solid lines represent the type of pattern created using 400, 600, 700, and 1,100 ms, and dashed lines represent the type of pattern created using 400, 500, 900, and 1,000 ms. (Color figure online)

Figure 3a shows that the average reproduction of intervals decreases from AS, to RS, and to DS. The mean reproduced interval was significantly affected by sequence type, *F*(2, 28) = 18.08, *p* < .001, ${\eta}_p^2$ = .56. The post hoc comparisons revealed that the mean reproduced intervals differed significantly from each other (*t*s > 3.2, *p*s* ≤* .02, *BF*s > 8.0), with AS having the highest mean (± standard deviation) (702 *±* 56 ms), DS having the lowest mean (660 *±* 55 ms), and RS falling in between (680 *±* 57 ms).

We further analyzed the reproduced intervals in the random sets based on the first interval being short (<700 ms), middle (700 ms) or long (>700 ms). Figure 3b shows a similar pattern to Fig. 3a, with the mean reproduced interval decreasing as a function of decreasing first interval duration (*long*: 694 *±* 57 ms; *middle:* 689 *±* 61 ms; *short*: 666 *±* 60 ms). The mean reproduction significantly differed based on the first stimulus in the random sequences, *F*(2, 28) = 7.78, *p* = .002, ${\eta}_p^2$= .36. Post hoc comparisons revealed that the *short* yielded significantly lower reproduced intervals compared with the *long* and the *middle* (*t*s > 2.59, *p*s* ≤* .02, *BF*s* >3*.0), while no difference between the *long* and *middle, t*(14) = 0.94, *p* = .36, *BF* = 0.38. The results suggest that the mean reproduction may be influenced by the first interval, rather than the sequential structure.

We conducted a similar analysis for the last interval in random sets with the same three categories (long, middle, and short), which revealed the mean reproduction did not differ, *F*(2, 28) = 1.20, *p* = .32, ${\eta}_p^2$= .08, *BF =* 0.38. Instead, the central tendency effects were different among three categories, *F*(1, 14) = 13.25, *p =* .002, ${\eta}_p^2=.49$. The mean slopes were 0.62, 0.50, and 0.31 for random sets ending with a *short*, *middle*, and *long* interval, respectively. Post hoc comparisons revealed that the slopes significantly differed from each other (*t*s > 3.40, *p*s < .013, *BF*s > 11). The central tendency was least prominent for random sets ending with a short interval and most pronounced for sets ending with a long interval, suggesting that the last interval influences the perceived variability of the sequence and subsequently influences the central tendency.

### Variance of time estimation

The standard deviations of the reproductions (Fig. 3c) were significantly influenced by both sequence type, *F*(2, 28) = 48.31, *p* < .001, ${\eta}_p^2$ = 0.78, and sample interval, *F*(6, 84) = 16.29, *p* < .001, ${\eta}_p^2$ = 0.54. Post hoc comparisons revealed that the reproduction variability was significantly higher for RS (*M* = 204.79) than for AS (*M* = 142.66) and DS (*M* = 138.72), *t*s > 7.4, *p*s < .001. However, there was no significant difference in variability between AS and DS, *t*(14) = 0.81, *p* = .43, *BF =* 0.35. The interaction between the sequence type and durations was also significant, *F*(12, 168) = 3.46, *p* < .001, ${\eta}_p^2$ = 0.20, partly due to the same interval having different positions in different sequences. The largest variability in the RS reproduction, significantly greater than the AS and DS reproduction, suggesting that the motor execution uncertainty was greatly influenced by the volatility. This was likely caused by unfamiliarity of executing irregular tapping.

### Estimates and prediction of the model

**Fig. 4:**
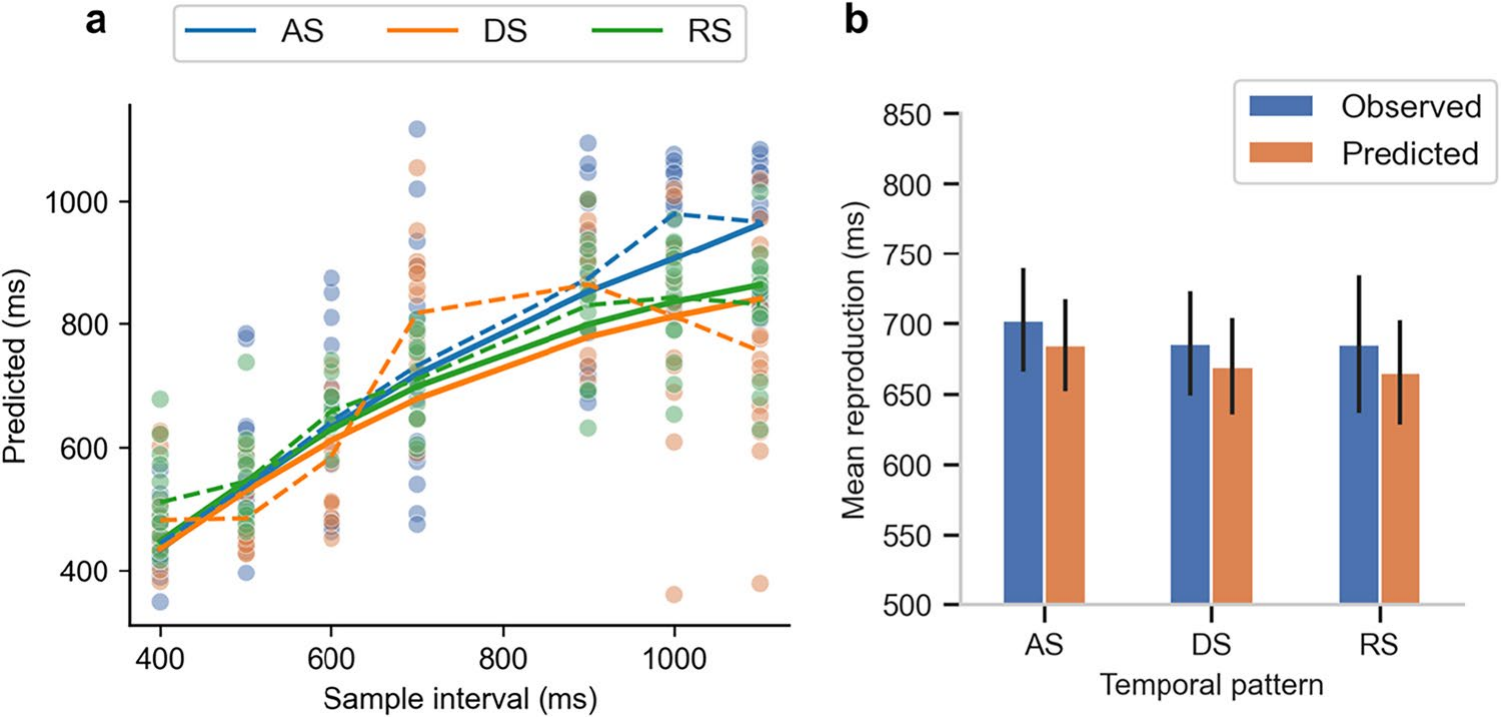
**a** Predicted reproduction durations of the model are plotted against sample intervals and the observed reproduction durations. Dots represent the observed data and dashed lines represent the averaged observed data across participants. Solid lines represent the predicted reproduction by the model. **b** Observed and predicted mean reproduction of the sequences as a function of sequence condition. Error bars depict one standard error. (Color figure online)

The Weber fractions of the sensory input and the mean prior (and associated *SD*s) were 0.18 ± 0.09 and 0.35 ± 0.03, respectively. The weight of the first interval was 0.195 ± 0.07, suggesting the first interval partially yet significantly influenced the mean prior. The volatility scaling factors were 2 ± 1.2 and 2.4 ± 1.4 for the AS and DS sequences, respectively, which is consistent with observed data showing the length of last interval may influence the perceived variability. With those parameters obtained from the AS and DS, the fitted volatility scaling for the RS sequence was 2.13 ± 0.17, in a similar range as the AS and DS. Figure 4a shows the model predicted mean reproductions (solid lines) versus the mean behavioral data (dashed lines). The mean coefficient of determination, *R*^2^, of the linear regressions were relatively high, 0.80, 0.62 and 0.91 for AS, DS and RS, respectively, which indicates the model in a good agreement with the observed data.

The model also predicted the order of the means and SDs of reproductions: the predicted mean and standard deviation for the AS was 684.9 ± 127.0 ms (observed = 702.5 ± 142.6 ms), for the DS 669.5 ± 132.7 ms (observed = 685.7 ± 143.8 ms), and for the RS 665.0 ± 143.9 ms (observed = 685.3 ± 190.1 ms). However, the model generated some minor undershot (Fig. 4b), likely originating from the model assumption. The model considered only the integration of the first interval and the veridical mean of 700 ms for the mean prior. As shown in previous research (Shi, Ganzenmüller, et al., 2013b; Wearden et al., 1998), auditory intervals are often overestimated, thus assuming the veridical mean (700 ms) is likely the primary source of this undershot.

## Discussion

The aim of this study was to investigate the effect of the sequential order of durations on temporal reproduction. The main findings were that, even when the sample intervals in the sequences were the same in terms of mean and variance, (i) the mean reproduced interval of a sequence was shorter with a sequence starting with a *short* relative to a long interval; (ii) the central tendency was more pronounced for sequences ending with *long* intervals; (iii) timing precision depended on the sequence structure, and the structured DS and AS were reproduced more precisely than the RS. Based on these findings, we proposed a pattern timing model that assumes the mean reproduction is a weighted average of the sample durations, with weights determined by their perceptual reliability. The central tendency arises from Bayesian integration of the ensemble prior and individual intervals. Our model was able to predict the observed behavioral temporal reproductions accurately.

Our perception of objects, sounds, or actions is heavily influenced by their surrounding spatial and temporal context (Schwartz et al., 2007). Several model frameworks, including Bayesian estimation models, have been developed to capture central tendency biases arising from trial history in time perception (Cicchini et al., 2012; Dyjas et al., 2012; Jazayeri & Shadlen, 2010). While standard Bayesian models can account for such biases by integrating the mean prior with sensory inputs (e.g., Jazayeri & Shadlen, 2010), they *fail* to consider the temporal order effects of probe stimuli, and hence fall short in explaining the findings observed in our study. Recently, Glasauer and Shi (2019, 2021, 2022) have investigated sequential-order effects of trials on magnitude reproductions. They found that high relative to low volatile sequences produce a stronger central tendency. Using an iterative Bayesian updating model, they could predict the differential central tendency effects and the sequential effects (depending on the previous stimulus) among different sequences. It is worth noting, however, that their studies focused on cross-trial sequential effects on an isolated trial rather than the temporal pattern within a short sequence. To the best of our knowledge, our study is the first to examine the sequence-order influences on temporal pattern reproduction.

It is essential to differentiate between individual interval and temporal pattern timing (Hardy & Buonomano, 2016), because interval timing refers to the timing of single durations, whereas pattern timing involves timing relationships among subintervals. When processing auditory interval patterns, assessing the statistical information of patterns is necessary in processes like speech and music (Paton & Buonomano, 2018). Humans can extract the mean frequency of a tone sequence (Piazza et al., 2013) and use statistical information of tone sequences in tasks such as speech categorization (Holt, 2006). In contrast to interval timing, it could be argued that participants adopt alternative weighting schemes to process subintervals of a pattern in pattern timing. For example, the first interval might engage more attention than subsequent intervals, as the onset often dilates time (Kanai & Watanabe, 2006; Rose & Summers, 1995). Indeed, the present study showed that the first interval in a sequence had a greater impact on the mean reproduced interval. Our model took this into account by adding the weight of the first interval. Thus, the current results emphasize that not only the statistical summary but also the order of individual durations in a temporal pattern contributes to the timing of those patterns.

Our study found that the central tendency bias in reproductions varied depending on the sequence structure. The AS had the lowest central tendency bias, while the DS and RS had similar and higher biases. The analysis of the last interval showed that sequences ending with a *long* interval had a higher central tendency bias. According to Weber’s law, the variability increases as the interval increases. Therefore, our findings suggest that the perceptual variability of a sequence was affected by its volatility and also by the recency effect—the last interval variability. Since the DS sequences had the longest ending interval, their perceived variability was overestimated, reaching a comparable level to that of the RS. It should be noted that perceptual variability is different from reproduction variability. Our analysis unequivocally showed that the RS had the highest reproduction variability, indicating that the volatility of a sequence, not just its physical variance, contributed to reproduction variability.

Previous research on central tendency bias (Jazayeri & Shadlen, 2010; Petzschner et al., 2015; Shi, Church, et al., 2013a) only considered the dispersion of the sampled intervals, while our study took sequential volatility into account. Our findings highlight the significance of considering sequence order in temporal reproduction. Our temporal pattern model, which included simple volatility scaling, accurately captured the order of variability across the three types of sequences. However, our model did not consider how volatility affects the uncertainty of motor reproduction, such as motor execution of irregular sequences. Future research on how sequential structure influences motor uncertainty could provide further insights into the reproduction of temporal patterns.

In sum, the current study highlights how the sequential structure of a temporal pattern influences listeners’ perceived ensemble mean and volatility, reflected by the average reproduced interval and the central tendency bias of duration sequences. The mean reproduction is largely influenced by the initial interval (i.e., onset dilation), while the central tendency effect is influenced by the volatility of the sequence. Moreover, timing precision of same durations differ depending on sequential structure, as it was shown by the lower precision of the random sequence than the structured ascending or descending sequences.

## Data Availability

The data supporting the findings of this study and the statistical analysis code used in the manuscript are available online (https://github.com/msenselab/temporal_patterns).

## Footnotes

1 Of note, the volatility should not be equated to the variability. Volatility measures the degree of unpredictable changes from one item to the next in a sequence, while the variability measures the dispersion of the sampled stimuli regardless of the sequential structure.
